# Legislation

**Published:** 1900-03

**Authors:** 


### LEGISLATION.

*Iowa.* This State has entered the field and passed through the lower branch of the Legislature a bill regulating the practising of veterinary medicine, surgery and dentistry.

Sections 1, 2, and 3 provide for the registration of non-graduates who have been practising for a livelihood for five years preceding the passing of the acts and those having diplomas from a legally chartered and authorized veterinary college. Registrations of non-graduates to be made before January 1, 1901, which registration to be approved by ten reputable freeholders and stockowners.

Board of Examiners to be three veterinarians, and to be appointed by the Governor, who shall be qualified residents of the State and in good standing in their profession. No representative of a veterinary college of the State shall be eligible to such appointment. At least one examination shall take place each year, others to be provided for by the board. All registrations shall take place through said board. Payment of registration fee five dollars. After January 1, 1901, all persons not before licensed shall pass an examination before the board, qualified by a diploma from a recognized school, and pay the examination fee of fifteen dollars. The board shall keep a register of all qualified practitioners in the State. Expenses are provided for in Sections 11 and 12 to any members thereof, the County Attorney of each county to conduct each transaction. The use of title without being authorized is punishable, also every practitioner of the State shall renew his license each year, through the board, by one dollar registration of the same. All expenses to be met by appropriation of its income for such purposes.

